# A Model-Based 3D Template Matching Technique for Pose Acquisition of an Uncooperative Space Object

**DOI:** 10.3390/s150306360

**Published:** 2015-03-16

**Authors:** Roberto Opromolla, Giancarmine Fasano, Giancarlo Rufino, Michele Grassi

**Affiliations:** Department of Industrial Engineering, University of Naples Federico II, Piazzale Tecchio 80, Naples 80125, Italy; E-Mails: giancarmine.fasano@unina.it (G.F.); giancarlo.rufino@unina.it (G.R.); michele.grassi@unina.it (M.G.)

**Keywords:** model-based algorithms, pose acquisition, uncooperative target, template matching, point cloud, LIDAR

## Abstract

This paper presents a customized three-dimensional template matching technique for autonomous pose determination of uncooperative targets. This topic is relevant to advanced space applications, like active debris removal and on-orbit servicing. The proposed technique is model-based and produces estimates of the target pose without any prior pose information, by processing three-dimensional point clouds provided by a LIDAR. These estimates are then used to initialize a pose tracking algorithm. Peculiar features of the proposed approach are the use of a reduced number of templates and the idea of building the database of templates on-line, thus significantly reducing the amount of on-board stored data with respect to traditional techniques. An algorithm variant is also introduced aimed at further accelerating the pose acquisition time and reducing the computational cost. Technique performance is investigated within a realistic numerical simulation environment comprising a target model, LIDAR operation and various target-chaser relative dynamics scenarios, relevant to close-proximity flight operations. Specifically, the capability of the proposed techniques to provide a pose solution suitable to initialize the tracking algorithm is demonstrated, as well as their robustness against highly variable pose conditions determined by the relative dynamics. Finally, a criterion for autonomous failure detection of the presented techniques is presented.

## 1. Introduction

The problem of autonomous pose determination of an observed object, relying on the data acquired through active or passive electro-optical (EO) sensors, has been widely studied in the fields of computer vision and on-ground robotics. Indeed, several applications can be found in the literature, like pose estimation of human heads [1] or hands [2] for surveillance and human-robot interaction purposes, or recognition and pose estimation of three-dimensional (3D) objects [3,4].

The capability of providing fast and accurate pose estimates is of high interest also for many space applications in which a servicing spacecraft (chaser) is required to maneuver in close-proximity of another space vehicle (target) to perform rendezvous, fly around or docking. Meaningful examples of these applications are active debris removal [5,6] and on-orbit servicing [7]. In these missions the pose determination task can indeed be extremely challenging, due to the fact that, in most cases, the target is totally uncooperative, being both unable to communicate with the chaser (absence of a data-link) and not equipped with easily detectable artificial markers on its surface. In this context, a possible solution for autonomous pose determination is to process the dataset provided by the EO sensor with purposely developed model-based algorithms which exploit a target 3D model, stored or built on board. However, the development of such algorithms in space applications is a quite demanding task, due to the limited resources available in terms of data storage and computing power, if compared with similar activities on ground.

Generally speaking, model-based algorithms are able to compute the relative attitude and position parameters by comparing global or local target features, as extracted from the sensor measurements, to the same ones in its 3D model [8]. Different types of techniques can be used depending on their role within the pose determination process. Specifically, a distinction exists between the acquisition step, which is performed when no prior knowledge of the target pose is given, and the tracking one, which relies on one or more previous pose estimates and is initialized by the acquisition step. In both cases, another relevant classification criterion derives from the type of input data required by these algorithms, which affects the selection of the EO sensor in charge of pose determination. Basically, monocular techniques can be applied to process images acquired by a single camera, while 3D ones exploit data that can be provided by active Light Detection and Ranging (LIDAR) systems or equivalently by passive stereo-cameras. In this latter case, the sensor output is a 3D point cloud which reconstructs the scene.

In this paper, the attention is focused on the pose acquisition step. Both in the case of monocular and 3D algorithms, pose acquisition is performed by comparing the acquired dataset to a reference database, which is built off-line, and whose elements (templates) correspond to specific sets of pose parameters. This approach is referred to as Template Matching (TM), since the pose estimate is obtained by finding the template which gives the best correlation when matched with the measured dataset. This estimate can be considered adequate, even if coarse in terms of accuracy, as long as it can successfully initialize the tracking step and is provided quickly enough to enable real-time operations. Hence, for pose acquisition, major issues are computational time and processing resources, as well as the amount of data to be stored on board, rather than pose accuracy. These issues have to be considered when investigating the applicability and effectiveness of TM in space applications. 

Different TM solutions can be found in the literature, which are able to deal with the computationally heavy search in the full 6-Degree-of-Freedom (DOF) database. In the case of the monocular approaches, a possible solution is to build the database of images with a hierarchical structure in which similar views are clustered at the lower levels of the hierarchy [9,10]. A similar approach, in the case of 3D techniques, is adopted by the polygonal aspect hashing algorithm, which limits the search space to the set of poses that allow the reference model to have at least partial overlapping surfaces with the input data [11]. A different approach, specifically designed for elongated targets, is to reduce the size of the database by splitting the estimation of the pose parameters in two phases: firstly, the two angles which identify the target main axis are determined by exploiting the principal component analysis, and, secondly, a 3D binary TM algorithm is applied to a 4-DOF database to compute the remaining rotation parameter and the relative position vector [12]. All the previously mentioned approaches exploit off-line processing to build and organize the on-board database, and they are aimed only at reducing the computational cost of the on-board processing. 

In this context, this paper presents a customized on-line 3D TM algorithm whose goal is to reduce the time needed for pose acquisition, while simultaneously cutting down the amount of data storage. This aspect is crucial for short-range operations. This is done by restraining the pose search space to a 3-DOF database consisting only of the attitude parameters, and building the templates on-line, just before correlating them with the acquired dataset. Moreover, an accelerated version of the presented technique is proposed which aims at further reducing the computational time. 

The purpose of this work is to demonstrate the effectiveness of the proposed techniques for pose acquisition of an uncooperative target, by analyzing their performance and robustness against highly variable conditions in terms of target relative position and attitude. To this aim, numerical analyses are carried out within a realistic simulation environment which provides the capability to reproduce the target model, various target-chaser relative dynamics scenarios, with particular concern to close-proximity flight, and the operation of an EO sensor able to provide 3D point clouds. In this respect, the attention is focused on active LIDAR systems since they present higher robustness to the variable illumination conditions typical of the space environment, and easier segmentation capabilities with respect to passive sensors, although they have higher mass, cost, power consumption, and lower frame rate [6]. Specifically, among the different solutions available for these systems [13], a scanning Time-of-Flight (TOF) LIDAR is considered. It is important to point out that the simulations are performed selecting a coarse angular resolution of the LIDAR, since it is of interest to verify the effectiveness and robustness of the proposed algorithms in processing sparse point clouds. Indeed, this allows reducing the computational burden and the time needed to complete the pose acquisition, which is a critical issue especially in close-proximity flight operations.

This work is realized in the framework of a feasibility study for an active debris removal demonstration mission [14], and the paper is organized as follows: Section 2 provides a detailed description of the 3D on-line TM algorithm as well as of its faster variant. Section 3 contains information about the target model, as well as a description of the simulators of the LIDAR measurement and the target-chaser relative dynamics. Finally, Section 4 comprises the selection of the simulated sensor parameters, the description of the simulation scenario and the results. It also includes the definition of a criterion for autonomous failure detection of the presented techniques.

## 2. Template Matching for Pose Acquisition

**Figure 1:**
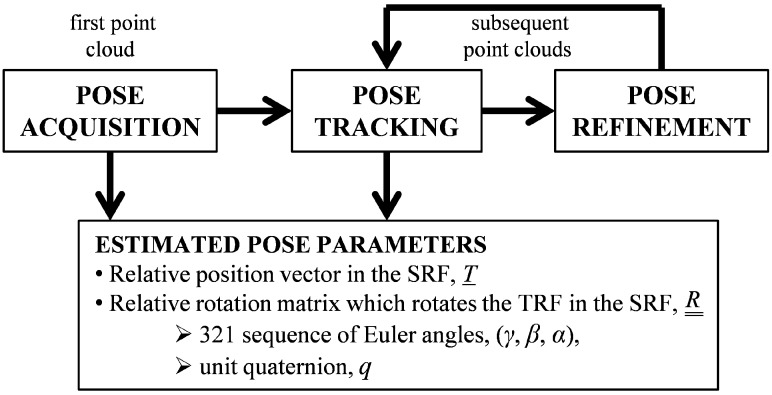
Logical scheme of the process for pose determination of a moving object.

The logical scheme used to estimate the relative attitude and position of a moving object is represented in Figure 1, where a scanning TOF LIDAR is the EO sensor providing 3D point clouds. The “pose acquisition” block receives the first acquired dataset in input, and then estimates the target pose without requiring any initial guess. Its output is then adopted to initialize the “pose tracking” block which estimates the time evolution of the pose parameters. Pose tracking is augmented by a “pose refinement” step, which allows improving the accuracy of the initial pose estimate provided to the tracking algorithm [15]. In this paper, the following rules are adopted concerning the mathematical notation: italic type is used for scalar quantities and quaternions, italic type with a single underline is used for other vectors, and italic type with double underline is used for matrixes. As regards the parameterization of the relative position and attitude of the target reference frame (TRF) with respect to the sensor reference frame (SRF), *T* is the relative position vector of the target with respect to the sensor, expressed in the SRF; while $\underline{\underline{R}}$ is the rotation matrix aligning the TRF to the SRF, which can be represented by both a 321 sequence of Euler angles (*i.e.*, yaw, *γ*, pitch, *β*, and roll, *α*) and the unit quaternion *q*.

This paper focuses its attention on TM-based techniques. The TM approach derives from the concept of searching, within an image (2D or 3D), for specific features and/or specific image sections, which can be matched to an assigned template [16]. The template can have the same size as the image, or it can occupy only a limited image area, while the matching function is carried out by exploiting a correlation approach. Specifically, different kinds of correlation laws exist, among which the sum of absolute differences [17], the normalized cross correlation [18], and the distance transform [19] (in case of feature-based TM) are most commonly used. In the framework of pose determination tasks, the TM techniques consist in building a 6-DOF database (*i.e.*, including all the relative position and attitude parameters), and using a correlation function to establish the degree of similarity between its elements and the acquired dataset. The pose solution is given by the set of parameters corresponding to the template for which the correlation function is optimized (*i.e.*, maximized or minimized). A specific feature of traditional TM algorithms, used for pose acquisition, is that the templates are generated during a preprocessing learning stage carried out off-line.

### 2.1. 3D On-Line Template Matching

Here, pose acquisition is entrusted to a customized 3D on-line TM algorithm which will be indicated below as on-line TM. The main innovative aspect of this technique is that the target relative position and attitude are estimated separately. Firstly, the centroid of the acquired LIDAR point cloud, ${\underline{\underline{P}}}_{SENSOR}$, is taken as the solution for the relative position vector. Then, the initial pose search is restrained to a 3 DOF database in which only the attitude parameters vary. This procedure provides a significant reduction of the number of templates to be generated and compared to the sensor data. Moreover, the database can be created dynamically, so that the only data to be stored on board are the geometrical information about the target needed to generate the templates. Hence, no off-line preprocessing stage is required.

**Figure 2:**
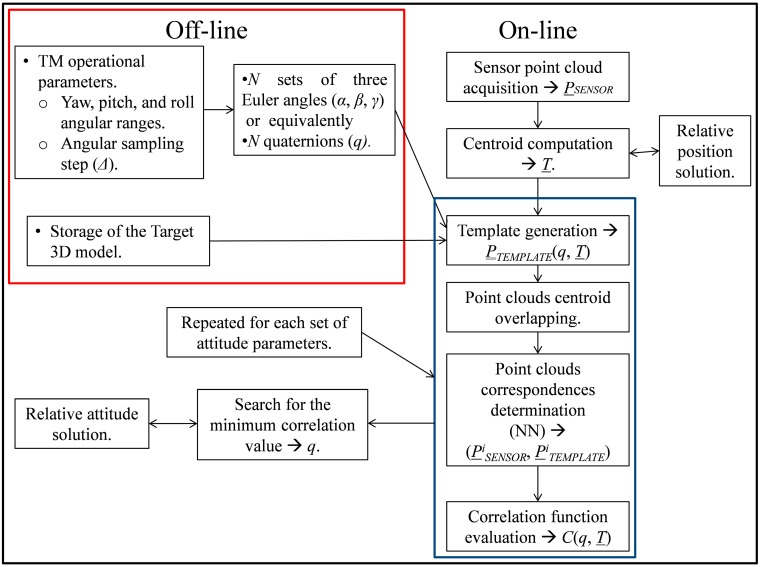
Flow diagram of the on-line TM algorithm.

A flow diagram which describes in detail the main steps of the on-line TM is presented in Figure 2. It is clear that, unlike traditional TM approaches, the only off-line actions (enclosed in the red box) are the definition of the algorithm operational parameters, and the storage of the target geometric model. Regarding the algorithm operation, it is firstly necessary to assign the range of variation of the Euler angles (*i.e.*, ]−180°, 180°[ for roll and yaw, and ]−90°, 90°[ for pitch), considering that the boundaries must be left out to avoid considering ambiguous triplets. Secondly, the angular sampling step (*Δ*) must be selected, considering that the lower the value of *Δ*, the larger the number of templates to be built on board.

Moving to the on-line stage, once the relative position vector is estimated as the centroid of ${\underline{\underline{P}}}_{SENSOR}$, a series of recurring steps, enclosed in the blue box in Figure 2, are performed for each set of Euler angles or, equivalently, for each corresponding quaternion. Specifically, a template (*i.e.*, a point cloud, ${\underline{\underline{P}}}_{TEMPLATE}$) is built by the simulator of the LIDAR operation (see Section 3), which exploits the available geometrical information about the target, and a correlation function with ${\underline{\underline{P}}}_{SENSOR}$ is evaluated. Specifically, a customized correlation function (*C*) is introduced in the paper, shown in Equation (1), which is defined as the mean squared distance of corresponding points betwee ${\underline{\underline{P}}}_{TEMPLATE}$ and ${\underline{\underline{P}}}_{SENSOR}$:

(1) $$C(q,\underline{T})=\frac{1}{N_{p}}\sum_{i=1}^{N_{p}}{\left|{\underline{P}}_{SENSOR}^{i}-{\underline{P}}_{TEMPLATE}^{i}(q,\underline{T})\right|}^{2}$$

In Equation (1), *N_p_* and *P^i^_SENSOR_* are, respectively, the size and the *i*-th point of the LIDAR point cloud, while *P^i^_TEMPLATE_* is the template point associated to *P^i^_SENSOR_*. It is clear that, in order to evaluate *C*, an intermediate step is required which consists in determining the correspondences between the points of the two data sets. Specifically, each sensor point is associated to the closest one in the template according to the Euclidean metric (Nearest Neighbor approach, NN). However, before performing point association, the two clouds must also be overlapped, *i.e.*, the template must be translated so that the respective centroids coincide. This is done in order to cope with the misalignment due to the fact that the templates are generated adopting a relative position vector whose estimation error can even be of the order of a few meters (depending on the size and shape of the point cloud). If not eliminated, this misalignment could easily make the algorithm produce wrong pose solutions. Once this procedure is repeated for each given set of Euler angles, the relative attitude solution is the triplet associated to the template which minimizes *C*.

### 2.2. On-Line Fast Template Matching

Although the proposed TM technique provides a significant reduction of the number of templates to analyze, it is still highly time consuming. So, a variant of the algorithm is introduced, indicated as on-line fast-TM, which aims at reducing the number of templates to be correlated to the sensor measurement. Specifically, the on-line fast-TM allows excluding those templates which are very badly correlated with ${\underline{\underline{P}}}_{SENSOR}$, *i.e.*, the templates producing large values of *C*. Indeed, they can be recognized *a priori* using the point cloud distribution, *D_BOR_*, computed as the mean distance of the cloud points from the LIDAR boresight axis, according to Equation (2):

(2) $$D_{BOR}(\underline{\underline{P}})=\frac{1}{N_{PC}}\sum_{i=1}^{N_{p}}\sqrt{{\left(x^{i}\right)}^{2}+{\left(y^{i}\right)}^{2}}$$

In Equation (2), *N_PC_* is the size of the generic point cloud, $\underline{\underline{P}}$, while *x^i^* and *y^i^* are the cross-boresight coordinates of its *i*-th point. So, referring again to the flow diagram in Figure 2, the on-line fast-TM requires executing the two final steps in the blue box only if a condition concerning the parameter *D_BOR_* is satisfied, as shown in Equation (3):

(3) $$\frac{\left|D_{BOR}({\underline{\underline{P}}}_{SENSOR})-D_{BOR}({\underline{\underline{P}}}_{TEMPLATE})\right|}{D_{BOR}({\underline{\underline{P}}}_{SENSOR})}\leq \tau$$

Equation (3) states that all the templates characterized by a distribution with respect to the LIDAR boresight direction which differs from the one of ${\underline{\underline{P}}}_{SENSOR}$ by more than a given threshold (τ), must be neglected. The threshold is determined by numerical simulations.

## 3. Simulation Environment

TM performance testing mainly aims at investigating the impact on performance of large pose variations, typical of debris monitoring and close-proximity flight scenarios. Since these conditions cannot be easily reproduced in a laboratory testing facility, a realistic simulation environment is developed comprising target modeling, sensor operation, and providing various relative dynamics scenarios.

### 3.1. Target Selection and Modeling

**Figure 3:**
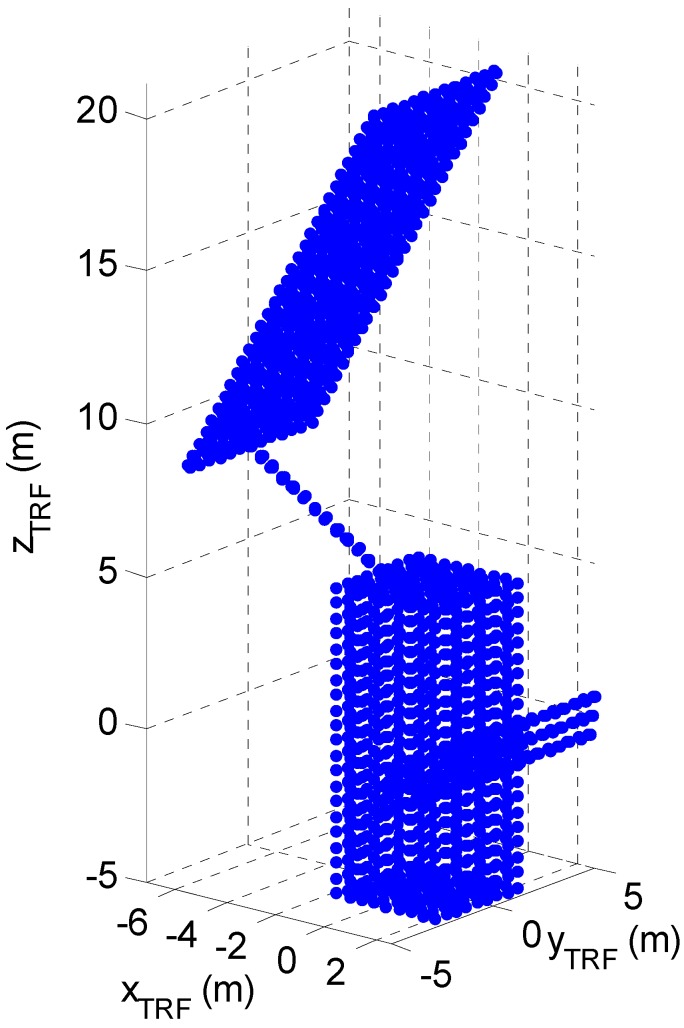
ENVISAT model point cloud represented in the TRF.

The on-line TM is designed to operate in the framework of space missions involving monitoring and close-proximity flight to an uncooperative space object. The simulations presented below are performed considering the satellite ENVISAT as target, since it is a perfect example of large debris in low Earth orbit. The assumed target 3D model, depicted in Figure 3 as a point cloud, is an assembly of five cuboid-shaped elements which represent the satellite main body, the solar array, the synthetic aperture radar antenna and their connective appendixes, whose dimensions are consistent with information provided in the literature [20,21]. As regards the surface characteristics, a mean value of 0.6 has been assumed for the reflection coefficient considering satellite surface optical properties degradation due to exposition to the space environment [22]. Specifically, we considered a highly reflective metallic material, *i.e.*, aluminum (ρ = 0.969), for the SAR antenna, a solar cell fused silica cover (ρ = 0.175) for the solar array, and, finally, a metallic material, *i.e.*, steel (ρ = 0.733), for the main body and appendixes. The TRF is a body-centered reference frame with the origin in the geometric center of ENVISAT main body. The actual ENVISAT geometry is more complex, and includes several devices and units installed on the external surfaces. However, using a simplified model can provide conservative results from the pose estimation point of view, since the presence of details on the external surfaces helps in distinguishing among similar and ambiguous poses. Geometric details also generate outliers because of multipath phenomena, which are included in the simulation as described in the following.

### 3.2. LIDAR Measurement Simulator

The simulations presented below are run considering a scanning TOF LIDAR. These systems are typically able to generate 3D point clouds by steering a pulsed laser beam with a couple of mirrors moved by high speed galvanometers [23]. The sensor operation is reproduced by a numerical simulator which requires the target-chaser relative position and attitude, the target model, and of course the LIDAR operational parameters in input. It is worth outlining that the deformation effects caused by the target relative motion while the sensor scans its Field-of-View (FOV) are not considered, since the relative dynamics is slow as compared with the LIDAR scan time, which typically is in the order of few centiseconds. The simulator is composed of the geometric, detection and noise models. The geometric model provides the ideal point cloud by determining the range of interception (*R*) of each laser beam with the target surfaces [15]. Once the ideal point cloud is computed, the LIDAR detection process is simulated in detail in order to establish whether the backscattered laser beams are detected or not. This can be done by evaluating the probability of detection (*P_D_*) of each received echo, as shown in Equation (4), as a function of the probability of false alarm (*P_FA_*) and the Signal to Noise Ratio (*SNR*) [24]:

(4) $$P_{D}=0.5\cdot \{1+erf[{\left(0.5+SNR\right)}^{0.5}-0.5\cdot \text{ln}\frac{1}{P_{FA}}]\}$$

The *P_FA_* is expected to be very low for laser ranging systems in close proximity operations in space, so it is set equal to 10^−4^. As regards the *SNR*, it is typically defined as the power ratio between signal and background noise. However, an alternative definition is here considered which consists in computing it as the ratio between the signal expected value and the noise standard deviation [25]. For a LIDAR system, the signal expected value is the average number of photoelectrons (*N_Sig_*) produced by the detector when a laser beam falls on its surface. This quantity is strictly related to the number of photons (*K*) hitting the detector during the integration time (*Δt*), which can be put equal to the laser pulse length (*τ_W_*) for this application [25]. However, these photons arrive at the detector at random times thus introducing an uncertainty in the value of *K*. This effect is known as photon counting noise and it can be evaluated by modeling *K* as a Poisson random variable whose mean, *E*[*K*], is equal to the detected energy divided by the energy per photon. Thus, *N_Sig_* can be computed by considering the quantum efficiency of the detector (*η*) which is a measurement of a device’s electrical sensitivity to light, *i.e.*, the ratio between electron generation rate and photon incident rate:

(5) $$N_{Sig}=\eta \cdot \text{E[K]}=\eta \cdot \frac{Pw_{DET}\cdot \tau_{W}}{h\cdot \nu }$$

In Equation (5), *Pw_DET_* is the backscattered laser beam power at the detector surface, *h* is the Plank’s constant and ν is the frequency of the electromagnetic radiation. *Pw_DET_* is estimated through the LIDAR-equation [25] based on the assumptions of well-resolved target (*i.e.*, the surface area which contributes to the target reflectivity is limited by the size of the illuminating beam rather than by the target dimension) and monostatic LIDAR configuration (*i.e.*, the angle of incidence and reflection are the same, *θ*, for any laser beam):

(6) PwDET(θ)=PwTRANPRF·τW·ρT·cos(θ)·D24·R2·τO

In Equation (6), *Pw_TRAN_* is the average transmitted power of a single laser pulse; *PRF* is the pulse repetition frequency; *ρ_T_* is the reflection coefficient of the surface hit by the LIDAR; *τ_o_* is the optics transmittance (typically an optical band-pass filter whose bandwidth, *Δλ*, is centered at the wavelength of the laser source), and *D* is the aperture diameter. As regards the noise standard deviation, the main phenomena affecting the performance of a LIDAR system are the laser speckle, and the thermal and background noises. However, the laser speckle effect is not considered here, since it is negligible for direct-detection LIDAR systems that use typically non-coherent laser sources (they aim at computing only the TOF) [25,26]. The thermal noise is a problem which arises at the detector level since any object at a temperature different from 0 K radiates photons. The variance of the number of thermal noise electrons (*Q_n_^2^*) depends on the detector temperature (*Temp*) and on the capacitance of the detector circuit (*Ca*) [25]:

(7) $$Q_{n}^{2}=\frac{k_{b}\cdot Temp\cdot Ca}{q_{e}^{2}}$$

In Equation (7), *k_b_* is the Boltzmann constant, while *q_e_* is the electron elementary charge. On the other hand, the background noise is related to the photons collected by the detector but not originated by the laser transmitter, which do not carry any information concerning the range to the target thus contributing to the system measurement noise. The total number of unwanted photoelectrons can be modeled as a Poisson random variable whose mean and variance (*N_b_*) can be obtained by adding the dark current contribution (*N_Dark_*) to the background ones (*Pw_BACK_*) [25]:

(8) $$N_{b}=\eta \cdot \tau_{O}\cdot \frac{Pw_{BACK}\cdot \tau_{W}}{h\cdot \nu }+N_{Dark}$$

In Equation (8), the main source of background photons is the sun, whose contribution is evaluated on the basis of the Standard Tables for Reference Solar Spectral Irradiances [27]. Once all the main sources of noise have been statistically modeled, the *SNR* denominator can be computed. By assuming that the overall noise is given by the simultaneous effect of multiple statistically uncorrelated terms, its standard deviation can be approximated as the square root of the sum of the variances of each individual noise contributions [25]:

(9) $$SNR=\frac{N_{Sig}}{\sqrt{Q_{n}^{2}+N_{b}}}$$

It is important to underline that if an Avalanche Photodiode (APD) is used as detector, then both *N_Sig_* and *N_b_*, inside Equation (9), must be multiplied by the APD gain (*G_APD_*). At this point, the *P_D_* can be computed, using Equation (4), and the detection process is completed by adopting a statistical approach which is basically a random extraction from a uniform distribution.

At the end of the detection process, the point cloud must be modified taking all the main noise sources into account. Specifically, the range and the Line-of-Sight (LOS) uncertainties are considered in this work. The former depends on the error in the TOF measurement and it is modeled as a Gaussian white noise (0, *σ_RANGE_*) superimposed to the detected range. As regards the latter, the angle between the simulated and the ideal laser beam directions is extracted from a normal distribution (0, *σ_LOS_*). Then, the simulated beam direction is rotated around the ideal beam of an angle extracted from a uniform distribution between 0 and 2π [28]. In addition to this, also the presence, of outliers is introduced within the acquired point cloud. They simulate possible multipath effects due to the actual target geometry and are extracted on the basis of a given outliers probability in the detected point cloud. These outliers are characterized by a range uncertainty whose standard deviation is four times larger than *σ_RANGE_* [11].

**Figure 4:**
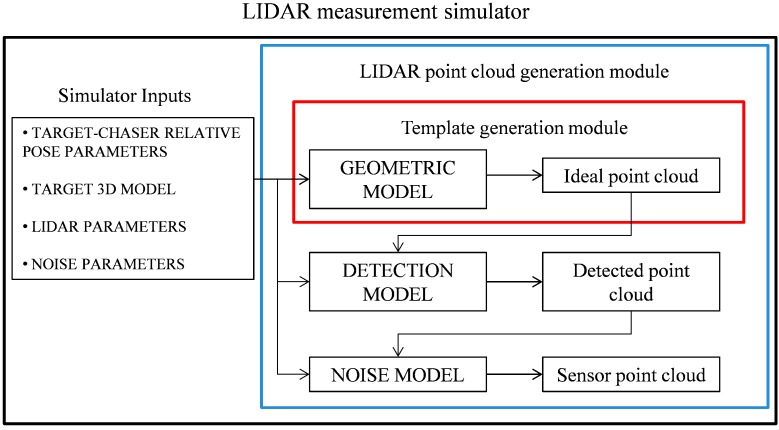
LIDAR measurement simulator: LIDAR point cloud and template generation modules.

The simulator structure is summarized in Figure 4. It includes two main modules: LIDAR point cloud generation (delimited by the blue box), which exploits all the models introduced above, and template generation (delimited by the red box), which is part of the on-board software implementing the proposed TM algorithms, and requires only the geometric model.

### 3.3. Relative Dynamics Simulator

The simulation environment allows reproducing different target-chaser relative dynamics scenarios according to the requirements of different close-proximity operations in space, e.g., rendezvous and docking, station keeping and target monitoring. This is done using a relative motion model which includes the secular effects of Earth oblateness (J2), and is based on a time-explicit formulation [29]. Specifically, this model is used to compute the differences in mean orbit parameters which satisfy specific design requirements of the relative trajectory. Once the relative trajectory has been defined, the target-chaser relative position and attitude parameters, needed by the LIDAR measurement simulator to generate the sensor point clouds are extracted by performing a uniform time sampling of its relative dynamics parameters.

## 4. TM Performance Analysis

### 4.1. Sensor Parameters

The simulated sensor spans a 40° × 40° FOV with 1-Hz updating frequency, and according to a raster scan pattern characterized by a fixed angular resolution (*δ_LOS_*) equal to 1° in both azimuth and elevation. This coarse angular resolution generates sparse point clouds of about 500 points on average. On the one hand this reduces the computational cost, on the other hand it makes the pose determination more challenging. All the parameters adopted to simulate the LIDAR transmitter [30,31,32], the LIDAR receiver [30,31,33] and the associated measurement noise [11], are selected considering typical systems adopted in space application. They are collected in Table 1.

### 4.2. TM Success Criterion

TM effectiveness is verified in terms of both computational cost and pose estimate performance. However, as regards this latter one, pose accuracy is not a meaningful metrics since the attitude parameter space is sampled with large angular steps (tens of degrees). In addition, the point cloud centroid, assumed as estimate of the relative position vector, can produce an error of the order of meters. Hence, a metric is introduced to establish if the pose acquisition can be considered successful or not. The criterion adopted here is to verify whether the pose tracking algorithm is able to estimate the pose with a prefixed accuracy level, starting from the solution provided by the TM. Pose tracking relies on the Iterative Closest Point (ICP) algorithm [34], in which two techniques for the matching step [15,35] are considered, namely the NN and Normal Shooting (NS). In conclusion, the TM pose solution is deemed successful if the subsequent ICP provides an attitude estimation error lower than a prefixed threshold. In this work, a 3-degree-threshold is adopted, since it represents approximately ten times the value of the ICP attitude accuracy level at regime [15,36].

**Table 1:** LIDAR system parameters.

LIDAR Transmitter Parameters		LIDAR Detector (InGaAs PAD) Parameters	
*λ*, laser wavelength	1540 nm	*η*, quantum efficiency	0.7247
*Pw_TRAN_*, average laser pulse power	1 mW	*G_APD_*, gain	10
*τ_W_*, pulse width	1 ns	*Ca*, capacitance	1.5 pF
*PRF*, pulse repetition frequency	10 kHz	*Temp*, operating temperature	273.15 K
*dθ*, beam divergence	0.02°	*i_D_*, dark current mean intensity	150 nA
**LIDAR Aperture Parameters**		**Measurement Noise Parameters**	
*D*, aperture diameter	2.5 cm	*σ_RANGE_*, range uncertainty	25 mm
**LIDAR Optical Band-Pass Filter Parameters**		*σ_LOS_*, pointing uncertainty	0.0007°
*Δλ*, filter bandwidth	24 nm	*P_O_*, outliers probability	5%–7%
*τ_O_*, filter transmittance	0.3898		

### 4.3. Simulation Scenarios 

**Figure 5:**
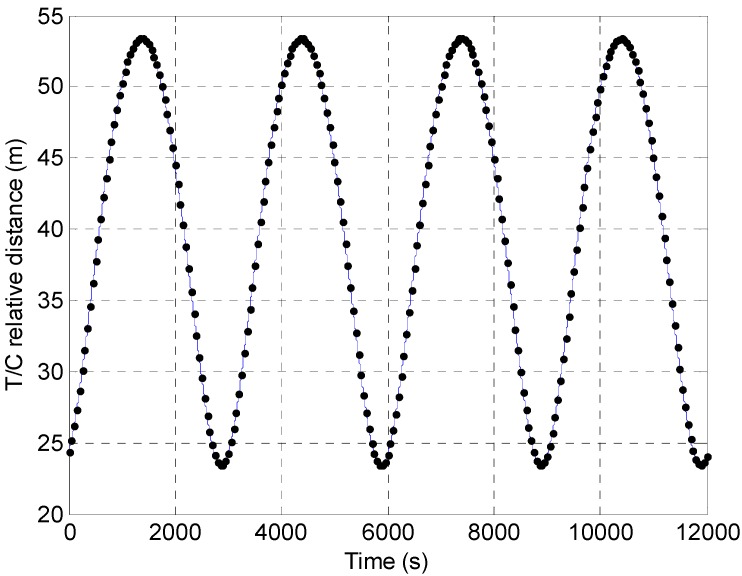
Time variation of the target-chaser relative distance along two consecutive orbits (in blue). The black dots indicate the time instants and the corresponding relative distances at which the TM algorithm is tested.

Two different scenarios are analyzed. Firstly, the algorithms’ success rate (*SR*) is computed considering different positions along a target-chaser relative trajectory designed for safe debris monitoring [36] using the simulator described in Section 3.3. This trajectory is a safety ellipse [37] in which the relative distance ranges from about 25 to 53 m. The evaluated *SR* is the percentage of successful TM pose solutions over 241 positions equally separated in time (50 s) along two relative orbits (see the black dots in Figure 5). The time step is selected so that two consecutive poses are significantly different in terms of both relative attitude and position.

The second scenario is introduced to deepen the results from the first analysis, and in particular to separate the effect of the target-chaser relative distance and orientation on algorithms’ performance. In this case, the *SR* is the percentage of successful pose solutions over a certain number of randomly generated sets of Euler angles, at different values of the target-chaser relative range, *i.e.*, at 20, 30, 40, and 50 m. Specifically, at each range, 500 sets of Euler angles are generated extracting yaw, pitch, and roll values from three uniform distributions.

### 4.4. Simulation Results

**Figure 6:**
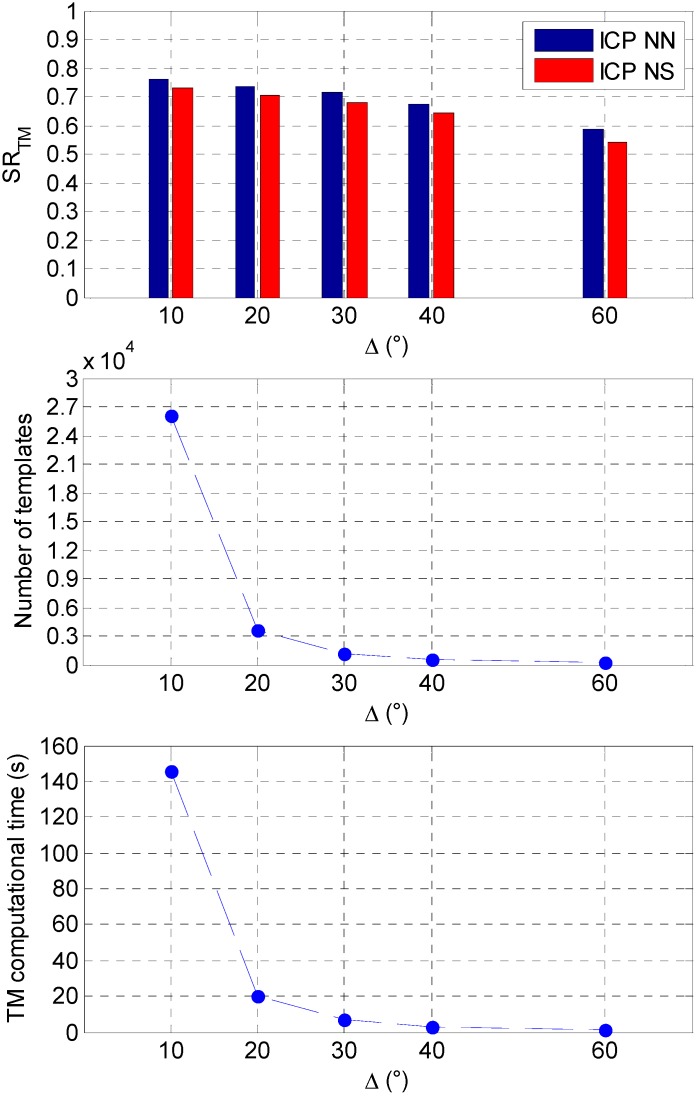
(**Top**) Effect of *Δ* on *SR_TM_* comparing the NN and NS approaches. (**Center**) Effect of *Δ* on the number of templates to be generated; (**Bottom**) Effect of *Δ* on the average computational cost.

The simulations are performed in MATLAB™ environment and run on a commercial desktop equipped with an Intel™ i7 CPU at 3.4 GHz. As regards the analysis over the relative trajectory, five different sampling steps (*i.e.*, *Δ* = 60°, *Δ* = 40°, *Δ* = 30°, *Δ* = 20°, and *Δ* = 10°) are considered. The effect of *Δ* on the on-line TM success rate (*SR_TM_*), expressed in the range (0, 1), as well as on the number of templates to be generated (and consequently on the computational cost) is shown in Figure 6, considering both the NN and the NS approaches for the matching step.

The first fundamental result is that the NN approach appears to be more effective than the NS one for the ICP matching step, at least immediately after the pose acquisition. This can be explained by remarking that highly rough initializations of the pose parameters (as the ones provided by the TM algorithm) can cause sensor-model point associations characterized by larger distances (if compared to the NN approach) when projecting a sensor point on the closest model surface, as requested by the NS logic. So, in the following, only the success rates achieved by the NN variant will be shown. As expected, a reduction of *Δ* produces an increase in *SR_TM_*, since it allows restraining the angular gap that the tracking algorithm has to compensate. Specifically, when *Δ* is 10°, *SR_TM_* reaches its maximum value of about 76%, but the number of templates is so large (~26,000) that the computational time also becomes unacceptably high (145 s) for close-proximity flight. On the other hand, if *Δ* is 60°, the number of templates drops down to 196 and so does the computational time (1 s), but at the same time the *SR_TM_* reduces to 59%. However, it is interesting to notice that the selection of intermediate values of *Δ* (*i.e.*, 20° or 30°) keeps the algorithm’s computational time low enough (20 s and 7 s, respectively) to enable real time operations, while simultaneously providing values of *SR_TM_* slightly higher than 70%. This can be explained observing that increasing *Δ* from 10° to 20° the number of templates reduces of one order of magnitude, while the average estimation error in the Euler angles (evaluated considering only the successful pose estimates) is approximately the same, as it is shown in Table 2.

**Table 2:** On-line TM average estimation error in the relative Euler angles averaged over the successful pose estimates within the two-orbit sequence of poses.

Average Attitude Estimation Error						
***Δ* (°)**		10	20	30	40	60
**Successful pose estimates**		184	177	173	163	142
**Euler angles**	**α (°)**	9.73	14.04	13.83	39.83	56.88
	**β (°)**	6.17	6.88	8.15	14.50	18.97
	**γ (°)**	21.40	22.70	37.45	47.62	78.45

By looking at the results in Table 2, it is possible to notice that the error in the yaw angle is always the largest. This can be explained considering that the simplified model used for ENVISAT has a preferential direction or symmetry axis. This makes the estimation of the rotation around this axis more challenging due to possible pose ambiguity issues. This situation is of course determined also by the fact that the model does not include many details present on the satellite external surface. Another important property of the on-line TM can be noticed by comparing the values of the estimation error in the relative position vector when the algorithm is successful to the same ones corresponding to algorithm failures. These results are collected in Table 3 where *T_X_*, *T_Y_* and *T_Z_* are the components of *T* in the SRF. When the LIDAR point cloud centroid is too far from the TRF origin (*i.e.*, the geometric center of ENVISAT main body) due to particular conditions in terms of target relative attitude, the relative position estimation error becomes too large, thus compromising the algorithm’s capability to find the set of sampled Euler angles adequately close to the real triplet.

**Table 3:** On-line TM average estimation error in the relative position vector components for the considered two-orbit sequence of poses.

Relative Position Vector Components		Average Position Estimation Error				
		*Δ* = 10°	*Δ* = 20°	*Δ = 30°*	*Δ = 40°*	*Δ* = 60°
**On-line TM success**	**T_X_ (m)**	2.809	2.697	2.689	2.772	2.853
	**T_Y_ (m)**	1.324	1.268	1.251	1.343	1.369
	**T_Z_ (m)**	1.924	1.936	1.868	1.808	1.868
**On-line TM failure**	**T_X_ (m)**	4.021	4.198	4.130	3.773	3.443
	**T_Y_ (m)**	1.588	1.714	1.732	1.478	1.412
	**T_Z_ (m)**	4.698	4.360	4.390	4.192	3.601

**Figure 7:**
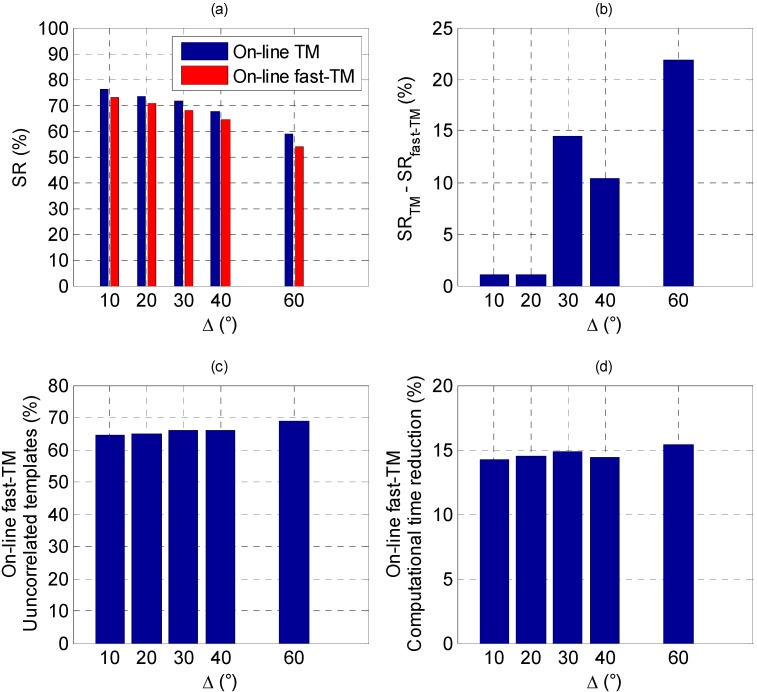
On-line TM *vs*. on-line fast-TM. (**a**) Success rate; (**b**) Loss of success rate of the on-line fast-TM; (**c**) Percentage of templates excluded from the correlation function evaluation; (**d**) Computational time reduction.

In Figure 7 the on-line TM algorithm performance is compared to its fast variant, defined in Section 2.1, in terms of both success rate (*SR_fast-TM_*) and computational cost.

Since a value of *τ* equal to 0.1 has been considered in Equation (3), the on-line fast-TM excludes from the evaluation of the correlation function, *C*, about 66% of the generated templates for any value of *Δ*, thus getting a reduction of about 15% in the computational time as compared to the basic approach. This reduction is limited by the fact that this technique has no impact on the time required for templates generation, which represents the main contribution to the overall computational burden. In terms of *SR*, although the decreasing behavior as a function of *Δ* is confirmed, the fast-TM strategy causes a loss of performance as compared to the basic approach due to the fact that, in some cases, it excludes also good candidate templates. However, if *Δ* is low enough (*i.e.*, 10° or 20°), the loss of success rate (*SR_TM_* - *SR_fast-TM_*) is extremely limited (about 1%), while it increases up to 22% when *Δ* grows to 60°. It is worth noting that when *Δ* is 40° the loss of success rate (10%) is lower than in the case of *Δ* equal to 30° (14%). This is related only to the particular sequence of poses analyzed over the relative trajectory.

**Figure 8:**
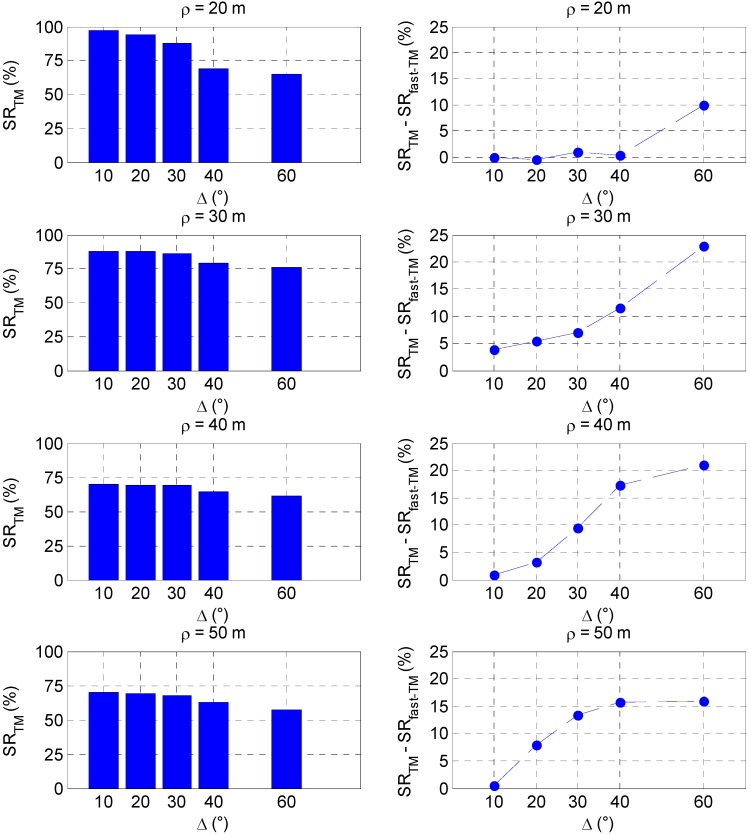
(**Left**) *SR_TM_* as a function of *Δ* at different ranges; (**Right**) Variation of *SR_fast.TM_*-TM with respect to *SR_TM_* as a function of *Δ* at different ranges.

The results about the success rate reduction derive from the fact that the condition introduced by Equation (3) is a reliable measure of the similarity between the templates and the LIDAR point cloud only if they contain enough information to perform the discrimination process. For instance, this happens when the number of templates is large (*i.e.*, when *Δ* is low), and the sensor point cloud is dense enough. To better explain this effect, the simulation results relevant to 500 randomly generated sets of Euler angles at different target-chaser relative ranges (ρ) are analyzed. Figure 8 shows the effect of *Δ* on *SR_TM_*, on the left side, and on *SR_TM_* - *SR_fast-TM_*, on the right side, while ρ varies from 20 to 50 m.

Again, a reduction of *Δ* produces an *SR* increase. More precisely, at ρ equal to 20 m, by changing the sampling step from 60° to 10° the value of *SR_TM_* varies from 65% up to 97%. On the other hand, at ρ equal to 40 m, by changing *Δ* from 60° to 10° the value of *SR_TM_* rises from 61% to only 70%. The effect of *Δ* on the algorithm’s performance weakens as the target-chaser relative distance increases since, being *δ_LOS_* fixed to 1°, the point clouds become so sparse (*i.e.*, their size reduces from about 490 points on average at 20 m to about 120 points on average at 50 m) that templates corresponding to pose parameters different from the actual ones can give rise to ambiguous matches (*i.e.*, they can produce similar values of *C*), independently of how well the attitude parameters space is sampled. Moreover, as the target moves far away from the chaser, the LIDAR *SNR* goes down, thus increasing the probability of point misdetection. This completely explains the worsening of the effectiveness of the proposed approach for pose acquisition at larger range. For instance, if *Δ* is equal to 30°, the value of *SR_TM_* goes from 88% at 20 m down to 68% at 50 m.

By focusing the attention on the right side of Figure 8, it is possible to notice that the increase of the range-to-target has a negative effect also on the *SR* performance of the on-line fast-TM. For instance, at 20 m range, the value of *SR_TM_* - *SR_fast-TM_* remains below 1% for Δ lower than 60° (which provides a loss of success rate of 9.9%), and, above all, by adopting the angular sampling step of 20°, it becomes −0.4% thus meaning that the adoption of the fast variant of the proposed TM algorithm is able not only to reduce the computational load but also to slightly improve the performance. On the other hand, at 30 m range, *SR_TM_* - *SR_fast-TM_* is always positive (never any *SR* improvement introduced by the fast variant), and gets worse for increasing *∆*, ranging from 3.9% to 23% for ∆ varying from 10° to 60°. Basically, this analysis over a wide range of pose parameters confirms that it is convenient to apply the fast-TM strategy only when enough information to solve the pose ambiguity problem is available, *i.e.*, at close range and/or selecting very low values of ∆.

As regards the computational load, also the time saving provided by the on-line fast-TM with respect to the basic TM algorithm is influenced by the variation of the target range. Specifically, as the target-chaser range enlarges, the size of the LIDAR point cloud tends to reduce. Hence, the contribution of the correlation determination task to the overall TM algorithm computational burden becomes less important, thus limiting the acceleration provided by its fast variant.

### 4.5. TM Failure Detection Approach

**Figure 9:**
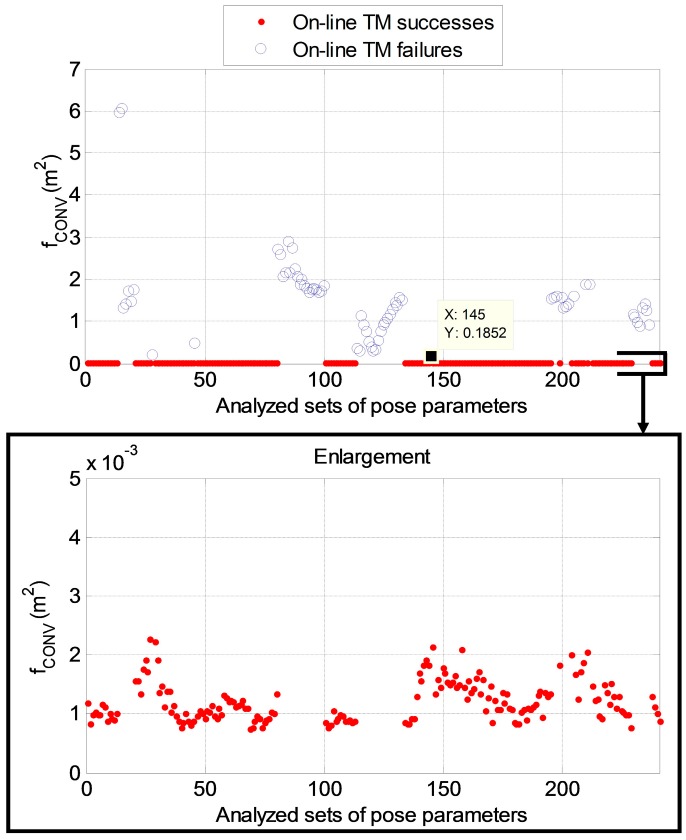
(**Up**) Value of the ICP cost function at convergence for the considered two-orbit sequence of poses; (**Down**) Enlargement of the same graph. The red dots correspond to successes for the TM algorithm, while the blue circles indicate the failures. The maximum value of *f_CONV_* which corresponds to algorithm failure is highlighted.

In order to cope with the cases of unsuccessful pose acquisition, a strategy is now introduced to autonomously detect the TM algorithm failure. To this aim, the analysis performed with *Δ* equal to 30° over the relative trajectory of Section 4.3 is considered. Moreover, the attention is focused only on the on-line TM algorithm. In this case *SR_TM_* is 72% and the computational time is 7 s. The strategy for autonomous failure detection is based on the value of the ICP cost function, as defined in [15], at the convergence (*f_CONV_*) of the tracking algorithm, that is at the first time instant subsequent to the pose acquisition. Figure 9 clearly shows that when the TM is successful the value of *f_CONV_* is of the order of few square millimeters, thus being significantly lower with respect to the case of TM failure where *f_CONV_* goes from more than 10 square centimeters up to few square meters.

This result can be generalized by analyzing all the simulation runs performed over the relative trajectory, as summarized in Table 4.

**Table 4:** Comparison among the values of *f_CONV_* in the cases of failure and success of the TM algorithm.

		*f_CONV_* (m^2^)			
	*Δ*(*°*)	TM Failure: Minimum Value	TM Failure: Mean Value	TM Success: Maximum Value	TM Success: Mean Value
**On-line TM**	**10**	0.1882	1.4620	0.0023	0.0012
	**20**	0.1882	1.4738	0.0023	0.0012
	**30**	0.1852	1.5309	0.0023	0.0012
	**40**	0.1539	1.3478	0.0026	0.0012
	**60**	0.1254	1.6681	0.0029	0.0013
**On-line fast-TM**	**10**	0.3086	1.5513	0.0023	0.0012
	**20**	0.3513	1.6901	0.1702	0.0022
	**30**	0.1254	1.4034	0.0023	0.0012
	**40**	0.1539	1.4273	0.0027	0.0013
	**60**	0.1254	1.7980	0.0026	0.0012

**Figure 10:**
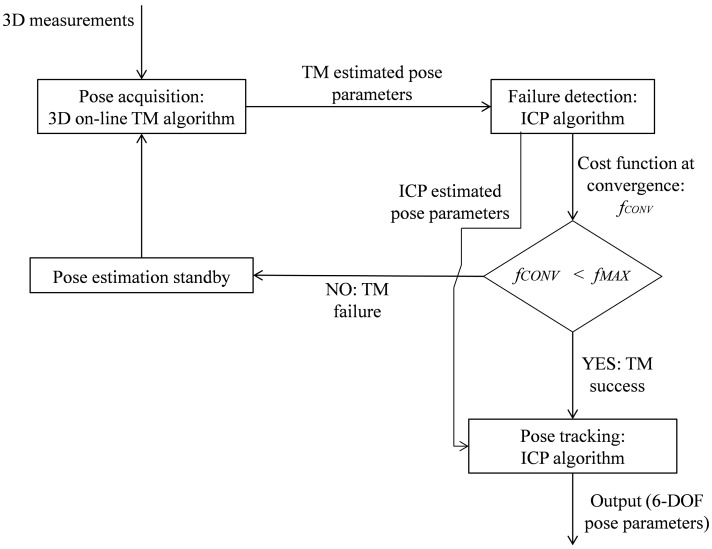
Logic for autonomous verification of successful pose estimation for the proposed TM algorithms.

A difference of three orders of magnitude exists between the average values of *f_CONV_* in the two cases. So, on this basis, it can be defined a maximum threshold value for the ICP cost function (*f_MAX_*) that allows the TM algorithm to autonomously detect its own failure. In case a value of *f_CONV_* larger than *f_MAX_* is obtained, it is advisable to wait for a certain amount of time and then to repeat the pose acquisition step until the condition for *f_CONV_* is satisfied, and the pose solution provided by the TM can be considered reliable for the tracking phase. The logic for the implementation of the proposed algorithms for pose determination during a close range relative navigation maneuver is shown in detail in Figure 10.

## 4. Conclusions

This paper presents a model-based template matching algorithm which exploits three-dimensional datasets, *i.e.*, point clouds, to perform the pose acquisition of an uncooperative target, thus being of relevance for extremely challenging space applications like active debris removal and on-orbit servicing. Specific feature of the proposed technique is the operation with sparse point clouds and a reduced number of templates built on-line. This allows not only limiting the computational cost but also cutting down the amount of on-board data storage. An algorithm variant is also proposed which allows a further reduction of the computational cost by limiting the search only to those templates which satisfy a similarity test with the sensor point cloud based on a purposely defined point cloud distribution metric. 

The proposed template matching techniques are numerically tested within a realistic simulation environment which reproduces target geometry and surface optical characteristics, the operation of a scanning time-of-flight LIDAR, as well as various target-chaser relative dynamics scenarios relevant to close-proximity flight operations. The simulation cases are designed to evaluate the capability of these techniques to provide an estimate of the pose parameters suitable to initialize the subsequent tracking phase, executed with different variants of the iterative closest point algorithm, namely nearest neighbor and normal shooting. Specifically, the template matching success rate is estimated with reference to a relative dynamics scenario relevant to a close-proximity flight, in which highly variable conditions in terms of relative attitude and position are realized, and to a set of randomly generated relative orientations at different target-chaser ranges. 

Results demonstrate that the success rate reduces for increasing angular sampling step and range, since they cause the algorithm to deal with limited number of templates and LIDAR measurements, respectively. On the whole, by working with about 1100 templates (*i.e.*, using an angular step of 30°), the success rate over a wide range of pose conditions is about 78% and the computational time is about 7 s, thus being suitable to close-proximity flight conditions. As regards the accelerated algorithm variant, simulations show that it provides a computational time saving of about 15% with respect to the basic approach, due to the fact that the search in the database is restrained to about 34% of the available templates due to the similarity test. However, this computational time reduction is coupled with a negligible loss of success rate (thus making convenient the implementation of this variant) only when the attitude search space is adequately sampled, *i.e.*, with 10° or 20° of angular step, or the point cloud is dense enough, *i.e*., at close range. These conditions provide indeed enough information to discriminate similar templates corresponding to ambiguous poses.

Finally, a criterion is introduced to autonomously detect possible failures of the template matching algorithm, deriving from the observation that the value reached at convergence by the cost function of the iterative closest point algorithm is on average three orders of magnitude larger when the template matching fails than when it succeeds. Simulation results demonstrate that this criterion could be used to increase the reliability and robustness of the proposed template matching algorithms for real-time on-board applications.

Further studies will be aimed at finding strategies to attain an additional reduction of the computational cost, while simultaneously keeping constant, or even improving, the success rate. This goal will be achieved by cleverly building the database, *i.e*., working at a consistent reduction of the template generation phase by exploiting information that can be inferred directly by the point cloud distribution. Moreover, the robustness of the autonomous failure detection criterion will be analyzed considering different targets in order to better focus the effects of target shape and size.
